# Patients’ perspectives of the feasibility, acceptability and impact of a group-based psychoeducation programme for bipolar disorder: a qualitative analysis

**DOI:** 10.1186/s12888-015-0556-0

**Published:** 2015-08-01

**Authors:** Ria Poole, Daniel Smith, Sharon Simpson

**Affiliations:** DECIPHer, School of Social Sciences, Cardiff University, 1-3 Museum Place, Cardiff, CF10 3BD UK; MRC/CSO Social and Public Health Sciences Unit, University of Glasgow, 200 Renfield Street, Glasgow, G2 3QB UK; Mental Health and Wellbeing, University of Glasgow, Gartnavel Royal Hospital, 1055 Great Western Road, Glasgow, G12 0XH UK

**Keywords:** Bipolar disorder, Psychoeducation, Group, Qualitative

## Abstract

**Background:**

Although there is some quantitative evidence to suggest the benefits of group psychoeducation for people with bipolar disorder, patients’ perspectives and experiences of group psychoeducation require in-depth exploration to enable us to better understand the feasibility, acceptability and impact of these interventions, the potential facilitators and barriers to engagement, and how to improve these interventions in the future.

**Methods:**

In-depth, semi-structured interviews were conducted with 13 participants of a psychoeducation programme for bipolar disorder in Wales, following their involvement in the programme. The data were recorded and transcribed verbatim and analysed using thematic analysis.

**Results:**

Findings demonstrate that group psychoeducation may impact on participants’ perceived social support, knowledge and acceptance of bipolar disorder, personal insights, attitude towards medication and access to services. Key recommendations for improvement included: allowing more time for group discussions, offering group sessions to family members and avoiding use of hospital or university venues for the groups.

**Conclusions:**

This is the first qualitative study of patients’ perspectives of a UK-based group psychoeducation programme for people with bipolar disorder, and findings present an in-depth account of how group psychoeducation may be experienced by patients. The recommendations for improving the content and delivery of group psychoeducation for bipolar disorder may enhance engagement and widen access to such programmes. Future research into psychoeducation for bipolar disorder should explore how to target and engage people of diverse ethnic backgrounds and those in lower socioeconomic groups who are less likely to access healthcare services.

## Background

Group-based psychoeducation for patients with bipolar disorder has been found to reduce recurrences of bipolar episodes [1, 2], time spent in a bipolar episode [1, 3, 4] and the frequency and duration of subsequent hospitalisations [1]. Severity of depression and mania may be reduced after one year following group psychoeducation for bipolar disorder [5], and after 2 years the severity and duration of manic symptoms may be reduced [6]. Studies have also indicated that social functioning and mental quality of life may also be improved by group psychoeducation [3, 4].

Qualitative studies examining one-to-one and caregiver psychoeducation for bipolar disorder provide a good starting point for understanding patients’ and caregivers’ experiences of psychoeducation and their motivation to undertake it, which may be beneficial for clinicians and for the development of psychoeducation interventions in the future [7–9]. However, there is a paucity of evidence for the benefits and drawbacks of group psychoeducation from patients’ and facilitators’ perspectives [10]. In-depth qualitative studies are vital to contribute to our understanding of how interventions are experienced by patients, relatives and those involved in their delivery. Such studies would enable exploration of the value of group psychoeducation from different perspectives and the barriers to benefitting from or undertaking the interventions, as well as delivering them in practice. It would also facilitate assessment of feasibility, acceptability and reach, and the personal impact of psychoeducation from patients’ perspectives, which is important for understanding the mechanisms of psychoeducation for bipolar disorder and also enhancing and promoting these interventions.

The Bipolar Education Programme Cymru, referred to as BEP-Cymru, was a manualised, group-based psychoeducation programme for people with bipolar disorder, delivered throughout Wales. BEP-Cymru was funded by the Big Lottery’s Mental Health Matters programme and was provided by the BEP-Cymru project team based at Cardiff University, who developed the intervention. The programme comprised ten group sessions delivered on a weekly basis to participants by two group facilitators, who have a background in psychiatry, mental health nursing or another related professional background and were trained according to the manual. There were no more than 15 participants per group. Sessions were two hours in duration and were structured in the following format: presentation, group exercise, refreshment break, facilitator led group discussion, and summary by facilitators. Its content was based on the psychoeducation programme by Colom and Vieta [11] and the Beating Bipolar internet-based psychoeducation programme [12]. Group sessions included the following topics: definition of bipolar disorder, causes of bipolar disorder, medication, psychological approaches, lifestyle, monitoring mood and identifying triggers, early warning signatures, and friends and families. We wished to explore the feasibility, acceptability and impact of this group-based psychoeducation programme for bipolar disorder from the perspectives of group participants. This research article describes the in-depth enquiry into patients’ perspectives of group psychoeducation for bipolar disorder.

## Methods

### Design and recruitment

This research was approved by the South East Wales Research Ethics Committee in May 2011.

Participants of the BEP-Cymru programme were invited to take part in the study via letter along with the patient information sheet and consent form. If prospective participants were interested in taking part in the study they had at least a week to contact the research team by telephone to ask any questions about the study or the nature of the data to be collected. RP obtained informed consent from those who wished to take part in the study at the introductory BEP-Cymru group session. It was made clear to patients that if they choose not to participate in the research study it would not affect their participation in the programme or their medical care in any way.

We obtained attendance records for participants of BEP-Cymru from the group facilitators to enable us to invite those who had attended the majority of the group sessions and also those who had dropped out, in order to understand their reasons for opting out of the programme. We purposively sampled participants on the basis of their level of engagement with the programme (identified by attendance records), their location (either North or South Wales, to minimise clustering effects) and the length of their diagnosis of bipolar disorder – to include participants recently diagnosed as well as those with a longer-standing diagnosis. RP conducted these interviews over the telephone at 3 months following their participation in the programme and audio recorded and transcribed the interviews verbatim. Interviews with patient participants were conducted until thematic saturation was achieved.

Qualitative interviews with participants explored their experiences of the programme, particularly in relation to its feasibility, acceptability and impact, and also complex processes and issues, such as motivations, decisions and outcomes, in depth and detail. It was anticipated that these personal accounts would provide insights into the acceptability and usefulness of the various aspects of programme, contextual factors, how the programme is received and its meaning for participants. Furthermore, the interviews aimed to capture any problems encountered with the implementation of the programme, the potential impact of the programme on participants and recommendations for improvement.

### Theoretical framework and analysis

The interviews were conducted flexibly and responsively to enable participants’ narratives to develop. Consistent with phenomenological theory, the primary focus of these interviews was on the nature and meaning of participants’ individual lived experiences, which were explored in relation to their personal contexts [13]. To explore the therapeutic mechanisms of BEP-Cymru we sought to examine the impact of the programme from participants’ perspectives and whether their experiences of the programme changed their perception of themselves and the disorder. Thematic analysis [14] therefore incorporated personal contextualisation, such as identity change and empowerment, amongst other emerging concepts relating to the feasibility and acceptability of the programme and its impact. Grounded theory was not deemed to be an appropriate strategy as it aims to obtain one core category – the essence of the findings – which becomes the theory to connect all the data [15]; whereas we wished to explore many aspects of participants’ experiences with a view to understanding the feasibility, acceptability and impact of the intervention, which are more specific and pragmatic concerns better suited to semi-structured interviews and thematic analysis. Interpretative Phenomenological Approach (IPA) was also considered as it is concerned with how people find meanings in their experiences; however, IPA questions are open and aim to explore a primary research question, rather than many, and the IPA sample is intended to be homogenous. Furthermore, because IPA analyses are very complex and in-depth, studies commonly become unmanageable if more than six participants are studied. We rejected IPA for this study because we wished to interview a heterogeneous sample and explore many factors which may have affected participants’ engagement with the programme, the acceptability of the programme and its potential impact. The interviews were therefore analysed using a thematic approach [16].

The interviews either began with an open-ended question about what BEP-Cymru was like for the participant or participants themselves initiated discussion of their experiences of BEP-Cymru after introductions. This format was to enable participants to feel more freely able to discuss their experiences and to their own agenda, as well as to our agenda. We felt that this approach empowered participants to discuss that which they felt most relevant or mattered most to them at the outset rather than waiting for the next question to come; however, we ensured that the interviews covered the feasibility, acceptability of the intervention, as well as how it may have impacted upon the participants.

RP recorded her impressions of the interviews immediately following each one, and developed these initial impressions when transcribing and familiarising herself with the data by mapping potential themes and categories. The data were inputted into NVivo 8 qualitative analysis software to code the data within an emerging thematic framework for themes which were developed and refined as analysis proceeded. The data were coded into categories, themes and sub-themes. Data saturation was deemed as having been reached at the point at which no new perspectives were being offered.

## Results

### Sample characteristics

Thirteen BEP-Cymru patient participants were invited to be interviewed and all took part. Of the 13 participants:

- 10 were female and 3 were male
- 7 attended a group in South Wales and 6 attended a group in North Wales
- 3 attended 1–2 group sessions; 3 attended 6–7 group sessions; 7 attended 8–10 group sessions
- 6 had been diagnosed for 2 years or less and 7 had been diagnosed for longer than 2 years
- Mean age 42.7 years (SD: 11.4)

Participants cited their reasons for deciding to participate in the group, which included: an interest in self-management, knowing nothing about bipolar disorder, being recently diagnosed with bipolar disorder, wishing to understand the condition better, seeking advice on how to live as normally as possible, to confirm or deny a diagnosis of bipolar disorder, to meet others with bipolar disorder, recommendation by their health care professional, and respecting the research at Cardiff University.

### Feasibility of BEP-Cymru

#### Mood affecting engagement with the course

Some participants (5/13) reported that their mood during the programme affected their ability to engage with it. Some participants (3/13) who reported feeling low during the programme felt apathetic, less sociable and struggled to concentrate. Others (2/13) who reported feeling high became angry, upset and found it difficult to sit still and focus. One participant reported that her mindfulness practice helped her overcome her low mood, and another participant reported being able to concentrate better when feeling high.

#### Timing of sessions

Two participants commented that mornings were difficult for them if they were feeling low and they would have preferred to attend afternoon sessions. Participants felt that evening sessions were preferable for those with day jobs, but some did not feel encouraged to leave the house on cold winter evenings.

*“[…] it’s dark at half past four and, you know, as I live alone as well, um the thought of going out and I don’t drive, can make you feel quite vulnerable, you know, so I am definitely affected by the weather”.*

*P2, female, South Wales*

#### Community venues preferred to hospital settings

Many participants (6/13) commented that they did not wish to attend the group in a hospital or university venue. It was important to them that the setting was neutral, sociable and central. Some (4/13) participants remarked that hospital premises had negative connotations for them and brought back memories of their bad experiences. Others (2/13) felt that the hospital was too far out of town to access.

*RP: “Have you any comments about the venue?”*

*P13: “I wouldn’t have it in the hospital.”*

*RP: “Right”.*

*P13: “Because of the different experiences that we’ve had, the bad experiences that most of us have had with the hospital. I think being attached to the hospital brings back bad memories for people […] being made to sleep in a […] blood spattered, food spattered wall, cell, like a prison cell was not conducive for improving one’s depression or bipolar or anything else quite frankly”*

*P13, female, South Wales*

All participants who were interviewed in North Wales highly praised the venues, which were both centrally-based community galleries. Participants commented that they were excellent, provided a lovely room and great refreshments, and were airy and light.

*“[…] the organisers made a conscious choice to find a space that was very airy and light and it was part of an Oriel and gallery here, in the middle of* [names location]*, um so these kind of, I felt like they really made a conscious decision about all these things, which everybody in the group appreciated it because bipolar people are very sensitive to their surroundings”*

*P5, female, NW*

#### Attendance at sessions

Participants provided a variety of reasons regarding why they missed sessions. Some participants (3/13) who felt low during the programme missed sessions because they were disinterested in the topic, felt unsociable, struggled to get out of bed or leave the house, lacked energy and felt fatigued. Others (3/13) missed sessions because of personal crises, hospital appointments or lacking the transport to attend. One participant reported missing sessions because the sessions were far from home and she didn’t feel motivated to make the journey on cold and dark winter evenings.

Participants gave many reasons for their attrition from the programme. Two participants in South Wales dropped out because they felt that the group was poorly facilitated in some cases. They complained that participants were sometimes allowed to rant off-topic. They also felt that the facilitators in some cases were patronising and lectured them and they felt pressure to contribute when facilitators asked questions around the room. Some participants (3/13) did not feel at ease within the group as a group member, or felt that the course was going over things they had already learned elsewhere.

Some participants (2/13) dropped out because of their low mood and lack of energy. Others (2/13) reported that they could not make that time of day because it clashed with their work schedule or because they felt that 10 weeks was too much of a personal commitment.

### Acceptability of BEP-Cymru

#### Content

Participants regarded the quality of the information presented within the course to be at an expert level, reliable and informative. They felt that the information was easy to understand, well-structured and sessions flowed well.

One participant remarked that the letter and supporting information which he received prior to starting the course was too formal in tone and he felt it was intimidating. He also commented that the information given was too vague and lacked an explanation about the format of the course.

*“I wasn’t sure if I was going to go or not you know, and I think perhaps the literature that arrived beforehand was a bit too formal (.) the letter that arrived first to say that you’ve been accepted on the programme (.) So perhaps it could’ve explained a bit more you know that there would be other people there, you know with the same sort of condition”*

*P6, male, North Wales*

Many participants (6/13) commented that they found the handouts to be concise and useful for remembering the course material or for catching up if a session had been missed. Two found the handout on debt and mental health to be particularly useful, and one participant commented that to receive a personal certificate and folder at the end of the course gave her a sense of achievement.

Some participants (5/13) reported that learning how to keep a mood diary was helpful, as was creating their lists of triggers and their action plan for becoming unwell. One participant said that creating his contacts sheet gave him a greater sense of security because he knew who to call in a crisis.

Participants enjoyed some of the exercises because they were fun and lightened the mood of the group. A couple commented that they particularly appreciated the visual representations within the exercises and presentations, particularly if they had literacy problems.

*“It was visual and it was mental as well, so it was a little bit of both, which was helped me because I can’t um, (.) I can’t write or anything properly, so the visual things, the visual work as well was better for me (.) the visuals on the laptop and things, you could see the life-chart and things on the laptop and then look at it as a group on a white board”.*

*P11, female, North Wales*

The session on medication was thought to be really helpful and the facilitators’ expert knowledge was appreciated.

*“[…] the medication session was very important because too often medication is given, especially by GPs and it isn’t explained, you know, for the actual side effects of these drugs and how important it is to take them regularly”*

*P6, male, North Wales*

Four participants said that they particularly valued the session on mood mapping, learning what was happening in a manic phase and learning to recognise their personal triggers for a bipolar episode. They found it helpful to inform their family members so that they could also recognise when their mood was becoming high or low. Others (2/13) appreciated the information within the session on lifestyle and took recommendations from the course. One participant also found the exploration of the link between bipolar disorder and alcohol problems useful.

One participant found the session on women with bipolar disorder, family planning and birth to be particularly helpful, and she shared the information with her friends with bipolar disorder who did not attend the course.

*“[…] in particular I was quite pleased with the session on women with bipolar and family planning and child birth and things (.) because I think that gets neglected quite a lot and that was like the first course I’d been to that covers that because it’s quite a complicated topic […] I shared the handouts that I had, um because I think it isn’t something that gets discussed that much, so I think that had, that was probably the biggest impact like for me and people I’m in contact with”*

*P12, female, South Wales*

Some (4/13) acknowledged that doing the life chart exercise had a psychological and emotional impact for themselves and others. Remembering upsetting times and losses as a result of the illness were hard for them to cope with and they felt depressed afterwards. Despite this, two participants commented that the constructing their life chart was helpful in that it confirmed when their illness began and enabled them to recognise former periods of mania or depression.

*“[…] it just brought up all the sadness really, what I was dealt, just a bad hand really, in what I was born into […] I went back the next week and the girl that was really depressed, I was worried about, she went back as well, she said she was really down and I said ‘I was that week, but it gives you stuff to think about, you know and you should use that time to discuss it with people who are close to you, people who are helpful and work through it.’ You don’t want to relive it all the time but it needs to be, if it’s that powerful, it needs to be faced head on and deal with it and just disempower it”*

*P9, female, South Wales*

#### Perceptions of the facilitators

##### Positive comments

Participants said the facilitators created a relaxed environment, involved everyone and balanced the formal with the informal aspects of the programme. They appreciated facilitators’ expert knowledge and said they answered their questions well.

*“I thought it was always good to have a psychiatrist in the room. Um, I think he researches bipolar so it was very good to have him there”*

*P1, male, North Wales*

Participants said that the facilitators “treated them like human beings”, were down-to-earth and participants felt accepted by them. Participants observed that the facilitators were keen to listen to them and learn from them. They appreciated the personal touch facilitators demonstrated in that they expressed an interest in them, were caring and looked after them.

*“Accepted, that’s one of the main things,* [F5] *was very good to just accept us the way we were and* [F6] *was really, really keen, he’s the psychiatrist, he works in* [names town/city] *I don’t know if you’ve met him, he’s recently started doing research as well and they were very keen to listen to us and learn from what we had to tell them, so to feel looked after and to feel like you matter, like you are relevant in this world, like you are important, that’s the sort of main thing they gave all of us”*

*P5, female, North Wales*

##### Negative comments

Some participants (3/13) criticised or complained about some of the facilitators because of their style of facilitation and poor group management skills. Some facilitators did not stick to time or address some participants’ offensive remarks.

*“I felt they just weren’t very good at managing the group really […] it got very boring to be honest, so I was kind of switching off and thinking about other things, um, there were people I know who, friends of mine who were going to the group who dropped out, that’s what they found, um that it was getting to the stage of being really unbearable and also I think when some people made remarks that were either a bit discriminatory or quite biased or even slightly offensive and they weren’t very good, they didn’t often, um kind of redress that or balance it out”*

*P12, female, South Wales*

Participants felt frustrated when people were allowed to talk about things that were not relevant to the day’s topic for too long. They felt that facilitators needed to summarise people’s points when they went off-topic because the rest of the group felt that they were not benefitting from the digressions. They also said that the facilitators did not ensure that everyone was given an equal opportunity to contribute to discussions and that some people were allowed to dominate the group.

A couple of participants felt that the facilitators were being patronising towards them which they found to be disrespectful and disempowering. They described feeling talked “at” rather than being talked “with”, and felt that their style was too didactic, lecturing or like classroom teaching.

*“My overwhelming memories and feelings of this course was ‘it’s them and us’ […] I didn’t feel very sort of ownership of ‘I’m finding out information for me to improve my health or my own management of my condition’ it felt more like ‘we’re telling you what you should do and if you don’t do it then you know on your own head be it’ […] just because we have bipolar it doesn’t mean we’re stupid or need lecturing at”*

*P10, female, South Wales*

Two reported feeling bored by facilitators’ dry and disengaging style of communication and did not feel engaged during the PowerPoint presentations. Some facilitators were also described to put pressure on people when asking questions around the room and some participants felt “put on the spot” or “trapped” with having to engage with uncomfortable topics. These participants felt that the facilitators did not appreciate the emotional impact of the course from participants’ perspectives.

Some participants (3/13) suggested ways in which group facilitation could be improved. They suggested that they be given time to be asked how they felt, to enable people to talk more and build group rapport and trust. One participant suggested that a more natural ice-breaker exercise where participants had more options regarding their responses, rather than answers to close-ended questions, would have felt more empowering. More time in the first sessions was needed for participants to get to know one another and gain an insight into why everyone was there. Participants felt this was important to feel safe, trusting and at ease with the group to facilitate openness and self-disclosure.

To prevent participants from going off-topic and also to enable them to discuss unrelated topics at an appropriate time, one participant suggested that facilitators operate a “parking zone” to make a note of topics participants wish to discuss in order to address them later.

Two participants said that facilitators could be more sensitive and flexible to the needs of the group and they would have appreciated hearing their points of view more than simply talking from the PowerPoint slides. They also recommended that the rules of the group should be negotiated with the group rather than just stated by the facilitators. They felt that this was important for the group to take ownership of the rules and personalise them, and also for developing trust within the group based on the understanding that others have engaged with the rules and have voiced that they would be respecting them.

#### Participants perceptions of the group experience

##### Positive comments

All participants appreciated the opportunity to share their experiences with other group members. They exchanged their experiences of their lives in general, their bipolar disorder, their health care, their health care professionals and their hospital treatment. They found this aspect of the course to be therapeutic, and learned from others’ insights and ways of coping.

*“It’s very difficult when you have bipolar to notice that you are going on a manic or on a depressive side, you don’t notice it yourself and to see other people talking about it about the behaviour that you have, you know, you start noticing things then during the day that you’re doing and you know, you can intervene then”*

*P6, male, North Wales*

Many (9/13) commented that they particularly appreciated being in a diverse group of people, some of whom were from different backgrounds, and reported feeling less lonely and isolated with the condition through meeting others with bipolar disorder. For some, the course provided their first encounter of meeting others who also had the condition.

*“Cos it is such a, it can be a very lonely a very isolating um condition. […] now I know what it is and I know other people in the same boat and I know how people are coping with it and stuff, whereas before I had no idea at all and I just felt so alone with it all, so yeah I’ve learnt a lot and it’s been a great help”*

*P4, female, North Wales*

Some participants (8/13) reported sharing knowledge with each other and offering advice. They also said that other members of the group were sensitive, concerned and respectful to how they were feeling.

Being a member of the group empowered participants who felt part of something important. Some (7/13) also commented that being with others with bipolar disorder and discussing experiences reduced the stigma they felt in day-to-day life.

*RP: “So, tell me your experiences of the BEP-Cymru group psychoeducation programme”*

*P5: “Oh it was really, really good, it was, well the best thing that’s happened since I’ve been diagnosed with bipolar, um, yeah it’s just so good to be together with like-minded people and feel like you’re normal because the rest of society makes you feel like you’re not normal, including most of our partners, who think we’re crazy […] we all felt stronger because we were in a group”*

*P5, female, North Wales*

*“[…] before I thought I was a bit mad in the past but now because I knew what it was and people had gone through the same experiences I realised that no, I wasn’t mad it was just part of the illness.”*

*P4, female, North Wales*

Participants commented on the importance of humour within the groups. Humour was used as a way of coping.

*P9: “[…] it was just nice to be in a room full of people who all had the same thread running through, but we’re so different, funny and warm and just to be able to discuss openly how this bloody awful illness had affected us. It’s priceless […]I’m terrible for making fun of myself and for making fun of my illness and psychiatry and the whole because I worked in psychiatry for a while and then I got ill and left […] Terrible, my sense of humour is just, lets me down really.”*

*RP: “Well does it or is it helpful in some ways?”*

*P9: “It’s my way of coping and that’s the way I am and I won’t change”*

*P9, female, South Wales*

For some, humour was used to “normalise” bipolar disorder.

*P5: “[…] I think the course really helped them to accept this is just who I am and we made lots of jokes about being bipolar and oh yeah all the stupid things we’ve done and (laughs)”*

*RP: “And did the jokes help?”*

*P5: “Yeah, loads, they give just a bit of light to the because you are treated as a problem like you said as well, with an illness and you’re not normal, all these things they are just really silly because we’re just human beings”*

*P5, female, North Wales*

Joking and talking in a light-hearted way was cited as being important for communicating within the group and increasing participants’ confidence.

Many (7/13) said that they really looked forward to the weekly meetings and were disappointed when they came to an end. They looked forward to seeing others in the group with whom they had developed camaraderie, trust and friendship. For some (4/13), attending the group on a weekly basis provided support when they were having difficulties and they described looking forward to the meetings as a way of “getting through the week”. One participant revealed that she missed the security of the group because it gave her an opportunity to meet with others with bipolar disorder, without which she lacked confidence to contact them.

*“I wouldn’t know how if I called somebody, I wouldn’t know how to talk to them, I haven’t got the confidence to text or to pick up the phone and say, ‘how you are, how are you or?’ I just keep myself to myself […] we have exchanged numbers but I haven’t used them and nobody’s called me either”*

*P11, female, North Wales*

For some participants (5/13) the groups enabled them to compare themselves with others who were “in the same boat” in having bipolar disorder. They found meeting with others to be helpful for gaining a perspective on the extent of their illness.

*“[..] when you’re in a group like that and you see some that are better than you, they’re doing much better than you, they’re in a better place and some are in a worse place and I don’t know it’s just like a measure really of how ill you are”*

*P9, female, South Wales*

A couple of participants said they were surprised and interested to hear that others had similar traumatic experiences to them.

#### Negative comments

It was the first time some participants had met with others with bipolar disorder, and they found the first meeting to be intimidating. Others (3/13) were dissatisfied by group members who were too dominant within the meetings and too focussed on their own agendas, and participants also reported feeling “put on the spot” with being asked to construct their life chart within the context of the group.

*“I’ve got friends I know who are doing it with their* [Community Psychiatric Nurses’] *and stuff, um like over a period of months because you know it can take a lot of time to be ready to look at what has happened throughout your life […]* [the facilitators] *did put a lot of pressure on people and then they kind of went round one by one and were kind of going, ‘oh, show us your life line and explain it’, and I thought that wasn’t very helpful because obviously some people were fine with it but apart from, if you hadn’t wanted to do it or you wanted to kind of start on your own but you didn’t really want to share it with the rest of the group, you didn’t really feel like you had an option. Um, so I think yeah that kind of being put on the spot I found quite pressurising.”*

*P12, female, South Wales*

One participant reported that she did not feel as though she connected within the group because it lacked others of a similar age to her.

### Contrast with other self-management courses for bipolar disorder

Participants who had previously attended other self-management courses for bipolar disorder compared their experiences of these courses with their experiences of attending BEP-Cymru. Two felt that the self-management course which was held over three days and offered by the Manic Depression Fellowship (MDF) was too intense. They felt that it was too introspective and did not adequately support vulnerable people who may have become traumatised by a distressing topic. One participant was particularly angered and upset by her impression that the MDF course focussed on certain problems and assumed that everyone within the group had the same experiences. She felt that they did not allow for divergent experiences or viewpoints within the group. Another participant felt that the MDF course had given her a better opportunity for sharing her personal experiences and learning from others’ experiences.

### Key recommendations for improving the course

Some participants (3/13) commented that they would have appreciated more information on certain topics and shared examples for further reading from self-help books. They felt that the course could have covered more on legal issues, psychological therapies, such as mindfulness and Dialectical Behavioural Therapy, and provided more up-to-date information on smartphone “apps” and mood diaries.

A couple also felt that the course was too medical in its focus. They commented that the focus on diagnosis and diagnostic categories was not helpful for practical self-management. They suggested that the course lacked the concept of self-management as a process which takes time, and would benefit from focussing on more practical and experiential considerations.

*“I think that was one of the things that was missing from the course was that idea of self-management as a process, um that it can take a lot of time for some people, or you can be good at one bit of it and it might take you time to develop other bits of it and I think that kind of time constraint on, you know,’we do this session, we do that next session, you know, are you much better at self-managing now?’ […] I didn’t really realise that until afterwards and I was kind of like, ‘hmm, hang on, you can’t do it all’, you know some people might but it’s a lot of stuff to go through”*

*P12, female, South Wales*

One participant suggested inviting someone with bipolar disorder to one of the sessions to relate their personal experiences and how they had managed their illness throughout their life.

Four participants suggested that they would benefit from the opportunity to involve their partners or relatives with the group. They suggested that family and friends who were concerned should be invited to a couple of sessions or that a session could be provided specifically for them. They felt that this may help them to understand and accept the illness more and gain an insight into it.

*“Strongly would recommend that partners, families were involved in at least two or three sessions, so that they have that understanding of what was going on because there’s nothing, no information for them at all […] To understand the moods because you are very, very hard to live with, so the partner does tend to back off and then you feel like you’re not getting any support when it’s not because you’re in a mood it’s because […] of the illness that makes you in that particular way but partners and family don’t have an understanding of it”*

*P4, female, North Wales*

Some (4/13) suggested that BEP-Cymru should arrange either a couple of recap sessions informally or a couple of casual meetings with or without facilitators after the course finishes. They stipulated that this should be organised by BEP-Cymru for participants even if a facilitator would not be present.

*“[…] perhaps something […] some months down the line that we came back to see how we are doing. […] I think it’s worthwhile, it’s such a good programme for it to end like that it needs something I think (.) it wouldn’t cost much would it to bring a group of people together every couple of months”*

*P6, male, North Wales*

Some participants (3/13) made recommendations for improving the life chart exercise. They suggested that it would be better to give participants the tools necessary for undertaking the task and provide examples of how to complete it. Participants needed to be better prepared for, and better supported with, the life chart exercise. Participants also recommended that the exercise should have a health warning and people should have the option not to do it if they don’t feel ready or they don’t want to do it.

### Reasons participants would recommend BEP-Cymru to others

Some participants (7/13) recommended the information they received on the course because it helped them to understand the illness better and was accurate and reliable.

*“[…] bipolar is like a foreign word really isn’t it? But when you get the information from you, you know what the disease is and what it involves you know”*

*P3, male, South Wales*

Many participants (9/13) would recommend the programme because it enabled them to meet others with bipolar disorder, discuss their experiences and make new friends from the group. They felt like they were no longer “the only one” with the illness and it had reduced their feeling of isolation. The course reduced the stigma they felt from having bipolar disorder.

*RP: “So how did you find the group experience?”*

*P4: “Recommend it to anybody […] because I’ve made friends, I’ve made friends from the group, um we meet on a Monday, we go for coffee or we go for lunch, we go swimming, it’s nice, yeah. Whereas before I didn’t have anybody, you know, I’ve lost lots of friends and stuff due to this illness and because they obviously don’t understand it and they couldn’t cope with my mood swings and things, so but at least you know with this group now, we’re all in the same boat, we all know how we feel and yeah it’s good”*

*P4, female, North Wales*

### Group versus computer-based formats

A few participants (3/13) reflected that an online course would be better if they were feeling low and were finding socialising difficult. Two found it hard to leave the house when they felt low and, therefore, would have preferred an online course at that time.

Many remarked upon the usefulness of being able to share experiences within a group setting, and some expressed reservations regarding online forums. Two chose not to use forums and one participant said she lacked sufficient computing skills to use a forum. Concerns surrounded the permanency of forum posts, a fear of feeling attacked or upsetting others.

*[…] because it’s open to a larger number of people, but at least in a group there’s like, you know ten people or whatever, but if you’re on a forum and you can have I don’t know how many people connected to it and they comment on something that you’ve said, it’s just kind of, I […] wouldn’t necessarily be asking or saying the things that I really wanted to say because I would be worrying that I might you know upset someone or get attacked by someone or something*

*P12, female, South Wales*

Some participants (4/13) opined that a computer-based course may be more accessible, especially for those who cannot attend a 10-week course due to other commitments or for those who cannot leave their home. They also commented that the forum would enable discussion and interaction with others.

Others (3/13) valued the opportunity to ask in-depth questions and receive an answer instantly within the group setting, which they felt a computer-based course would lack.

### Impact of BEP-Cymru

#### Impact on knowledge

Many participants (8/13) commented that they had a better understanding of bipolar disorder, the causes of bipolar disorder and its treatment. Some discovered that the illness was more complex than they had previously realised.

*“[…] it was nice to know what the cause of it was, yeah. Whereas before I didn’t have a clue, you know? I’d just like do crazy stuff and end up in hospital and not know why, um and just being diagnosed with is something and being able to learn about it was a huge help”*

*P4, female, North Wales*

Some (3/13) appreciated attending the course even though they were already familiar with the information presented because the course confirmed what they already know and revisiting topics was helpful for them. Others (2/13) reported their ability to explain their bipolar disorder in a concise way to others, without feelings of shame or stigma.

*“I can explain it quite concisely now and I know the points that people want to hear and need to hear, whereas before you’d be explaining something that sounds so bizarre, you know all these mood swings and things […] without feeling you have to hide anything”*

*P6, male, North Wales*

#### Impact on social support

The main reason participants cited for appreciating the course despite not learning anything new was the group experience. Participants particularly welcomed feeling part of a group with others who also have bipolar disorder and the opportunity to share their experiences, listen to others and offer support. They said that the openness of the group negated any shame or stigma associated with bipolar disorder, and the course enabled them to feel less isolated with the illness and become aware that others also suffered from bipolar disorder in similar ways to them. This realisation stemmed from others sharing their experiences of living with bipolar disorder and relating how they cope.

*“I didn’t feel so isolated, you know. I thought that there are other people who suffer in the same way that I do […] I benefitted a lot from talking about my illness with these people and finding that they shared aspects of the illness […] there were other people like me”*

*P7, female, South Wales*

The course did not impact on some participants’ personal relationships. This was because they felt that either their family did not need to be informed about their bipolar disorder or others were not interested or able to respond to them when they were unwell.

Some (5/13) said that their families wouldn’t be interested in reading hand-outs, although they would be interested in learning about bipolar disorder within a group session specifically for them. Others (2/13) described their partners to be more accepting and trusting of them and supported their involvement on the course.

Many (9/13) befriended other participants on the course, and one group continued to meet as a bipolar support group beyond their involvement in BEP-Cymru.

*“Just there for support you know, if someone’s not feeling well, somewhere where they can go where they’ve got support, because when you’re not feeling well it’s hard to actually get out of the house, whereas if you know there’s a group there who are going to support you, the group could actually pick you up and take you out, you know?”*

*P4, female, North Wales*

#### Impact on confidence, stability and acceptance

Some participants (3/13) commented that their confidence had improved as a result of attending the course and they were better able to cope with challenging situations. A couple reported that as a result of attending the course they felt calmer and their moods had become more stable. They said that they felt “normal” as a result of learning about the illness and feeling well.

*“I’m a normal person, I think normal thoughts, I don’t want to do anything too outrageous […] I’m calmer, I’m interested in reading books and things you know, things I wouldn’t have been bothered to do and I like watching certain programmes on television […] I’m interested in things that I wouldn’t bother with before, all I was interested in before is how I felt all the time, very miserable or very happy, such a change in my feelings that it was unusual, I didn’t understand it you see but if you have a long session with people who have got the disease, you can understand it, you can leave the room knowing a little bit about it”*

*P3, male, South Wales*

A few (4/13) reflected upon how the course had impacted on their acceptance of bipolar disorder. For some, the acceptance and respect they felt from the facilitators reduced the stigma associated with bipolar disorder.

*P5: “I think the fact that they are, were there as persons as human beings, as themselves and made us feel like we were human beings and could be ourselves”*

*RP: “So, I’m thinking did it reduce stigma for you then, is there a stigma around it?”*

*P5: “Yeah, massively, massively, yeah, yeah, I don’t know you should, you can’t experience it but you should have a go at telling people you are bipolar, wow! Just for a joke. You’ll get a feel of what it is like”*

*P5, female, North Wales*

#### Impact on access to services

Some participants (3/13) particularly benefitted from the contacts sheet which was created during the course and comprised numbers of services and individuals to contact in an emergency. This enabled them to access help when they needed emergency assistance.

“[F5] *had given us this […] leaflet where you can put all the numbers of the emergency in the home treatment and stuff and that’s, well that turned out to be like a life saver today because I managed to get through these numbers that* [F5] *gave me, I managed to get through to people who could help us”*

*P5, female, North Wales*

#### Impact on diet

One participant commented that as a result of the lifestyle module he became aware of the importance of a healthy diet and not drinking alcohol.

*“[…] in terms of my lifestyle issues, I’ve taken that on board in terms of healthy eating and no alcohol […] because that was a very good module, lifestyle factors […] I used to uh, try and control my condition by […] drinking alcohol and eating comfort foods and you know, the module just made it quite clear that you’ve really got to live a healthy lifestyle as possible um, to try and gain control so the medications can work”*

*P1, male, North Wales*

#### Impact on insight

Many participants (7/13) reflected that as a result of attending the course they could understand what bipolar disorder is and gain an insight into themselves. One participant commented that she is now able to recognise when she is experiencing a high or low episode.

*“I understand now when I am on a high and when I’m on a low, as before I didn’t”*

*P11, female, North Wales*

Some participants (4/13) commented that they were now able to accept their diagnosis, and realised that it was their personal responsibility to manage the illness and “keep on top of it”. They were aware of how to control their symptoms and maintain their wellbeing.

*“It’s made me realise that I do have bipolar (laughs), for a year I was thinking, ‘oh I don’t have this thing you know, it’s not me, sort of thing’, once you see or you meet other people you see you think ‘I do that’ or ‘I behave in that way, so, so it really makes you think you know, yes I do have this condition, I have to deal with it’, yeah”*

*P6, male, North Wales*

For some (3/13), a greater acceptance of themselves and their diagnosis resulted in less self-blame and more compassion towards themselves.

For one participant, however, attending the course had confirmed her belief that she has been misdiagnosed with having bipolar disorder. She describes herself as being naturally exuberant, which some health professionals have mistaken for mania, and believes she has only suffered from depression in the past.

*RP: “So how do you feel about your diagnosis of bipolar disorder now?”*

*P13: “Absolutely up your arse! Excuse my language”*

*RP: “So you don’t think you have bipolar disorder?”*

*P13: “I believe I don’t, no […] it’s not just me […] I mean the thing is I exhibit and that’s what my partner says […] a highly sensitive, highly principled, articulate, eloquent, whatever you want to call it, person, who is very, very motivated, very driven and very active. I mean I’ve done ballet, dancing, every day and I’m very, very busy, I always have been, except when I’ve been depressed and looking back I’ve done a mood chart, I’ve done a mood chart through my life and when I was on the course and I realised that I was only low when certain circumstances and people came into my life”*

*P13, female, South Wales*

Some (3/13) said that they could recognise what triggered their moods now.

*“I recognise my triggers, whereas before I wouldn’t, I’d get panic attacks and hyperventilate and get confused and run around dizzy and getting angry and not knowing what was going on but now I know what’s going on and why it’s going on”*

*P4, female, North Wales*

Others (2/13) realised that certain people or circumstances would trigger their low mood. They said that the course gave them an insight into previous failed relationships and one participant cut ties with certain family members who were not considerate to her mental health.

*P9: “[…] at the time there was a lot going on and there was a lot of trauma around my family and it wasn’t getting any better and I […] couldn’t take it anymore and I made a decision after the course and cut off from them and I’m better, I’m more well now, I should have done it years ago”*

*RP: “To cut off from your family who…?”*

*P9: “Well just a couple of them, not all of them, the ones that were making me ill really, year after year, the same things, just not being very considerate to my mental health”*

*P9, female, South Wales*

#### Impact on attitude to taking medication

Some participants (3/13) said that they had become less resistant to taking medication for bipolar disorder because they acknowledged that it enabled them to feel well, and they accepted that they would be taking medication for the rest of their lives to control the illness.

*“I could not accept I had to take tablets for the rest of my life, I just could not accept because the dosages that I was taking, they were so high and it was just accepting I had an illness, um I couldn’t cope with that at first but then after talking it through, you know, what basically what they were saying these tablets and stuff they help you manage it, they don’t stop it, they just help you manage it, control it better, so I’ve come to terms with the fact that yeah, I will have to take them for life, whereas before I couldn’t and (laughs) it was mixed emotions it was”*

*P4, female, North Wales*

Two participants re-evaluated their medication as a result of the session on medication. One participant remarked that because the facilitators had provided him with informed reassurance about his medication, now he takes his medication regularly as prescribed.

*“I was concerned that I was on Lithium, Quetiapine and an anti-depressant you know but they,* [P6] *said no this is normal and these drugs go together well, yeah it puts your mind at ease really these drugs aren’t as scary as you think. So I think I was going, I went for about two years without medication, um because I was concerned about taking, which I wouldn’t be now having gone to these sessions […] and I make sure that I take them regularly because they can, some of them can have an effect if you take them two or three hours late, you know on the next day for example”*

*P6, male, North Wales*

## Discussion

### Main findings

#### Overview of key themes

Table 1 highlights the key themes in relation to the feasibility acceptability and impact of the programme from the participants’ perspectives.

**Table 1:** Main areas of discussion arising from the qualitative interviews with BEP-Cymru participants

Feasibility of BEP-Cymru	
Mood affecting engagement with the course	
Timing of sessions	
Community venues preferred to hospital settings	
Attendance at sessions	
Acceptability of BEP-Cymru	
Content	
Perceptions of the facilitators	
Participants perceptions of the group experience	
Contrast with other self-management courses for bipolar disorder	
Key recommendations for improving the course	
Reasons participants would recommend BEP-Cymru to others	
Group versus computer-based formats	
Impact of BEP-Cymru	
Impact on knowledge	
Impact on social support	
Impact on confidence, stability and acceptance	
Impact on access to services	
Impact on diet	
Impact on insight	
Impact on attitude towards taking medication	

#### Feasibility of BEP-Cymru

Most participants were able to attend the course; however, venues which were located more centrally were easier for participants to access. Afternoon or evening sessions were preferable for some participants, especially for those with day jobs; however, courses held on dark winter evenings may inhibit participants who may feel vulnerable with using public transport at that time or reluctant to leave their homes. The type of venue was important for some participants who did not wish to attend courses in hospital or university venues and preferred light and attractive community-based venues.

Participants needed to feel well enough to be able to engage with the course, and those who were experiencing depression or manic symptoms were either unmotivated to participate or unable to concentrate on the sessions.

Reasons for dropping out of the groups included: dominating group members, feeling patronised by some facilitators’ lecturing style, feeling pressure to contribute to group activities, feeling they were not learning anything new or because of a lack of proper facilitation of the group. Other participants dropped out because they were depressed, they were unable to commit to a 10 week course or because the course clashed with their work schedules.

#### Acceptability of BEP-Cymru

The course materials and handouts were commended for their expert and reliable information. Participants particularly appreciated topics regarding medication, mood mapping, recognising triggers, lifestyle, and women with bipolar disorder; however, the life chart exercise was a concern for many participants because they felt unprepared for the psychological and emotional impact of the exercise, they felt pressurised to do the exercise, or they consequently felt depressed. Despite experiencing low mood after creating their life charts, some participants appreciated the opportunity the exercise had given them to review their history of the illness and in doing so they confirmed when their illness began. Participants recommended that future participants should be appropriately supported when constructing their life chart, advised about the psychological impact of undertaking the life chart exercise and given the option not to do it.

It was recommended that concerned relatives should be involved with the psychoeducation groups and it would be useful to provide sessions specifically for them to enable them to better understand the condition. Regarding continuation of the groups after the course, some participants suggested that occasional follow-up sessions be arranged to see how participants are getting on, either with or without facilitators and arranged by BEP-Cymru. Many felt that it was useful to share experiences within a face-to-face group and receive immediate and in-depth answers to questions from group facilitators.

Participants’ perceptions of the facilitators varied depending on whether they were based in North or South Wales. Facilitators were praised primarily for their accepting, caring and down-to-earth approach and for balancing the formal and informal aspects of the course, and were also commended for being informative and professional. Some facilitators were also criticised for not managing dominant group members, poor time management, a “lecturing” style and putting the spotlight on people when asking questions around the room. Some recommended that facilitators should: ask people how they are feeling and provide more opportunities for them to talk, allow more time for group members to get to know each other in the first session, present open-ended rather than close-ended questions for the ice-breaker exercise, ask participants to suggest and negotiate the rules of the group, talk more personally and conversationally rather than from lecture slides, and prevent dominant people from digressing off-topic by taking note and address their points later.

Participants stated the importance of sharing personal experiences, supporting others in the group and offering advice. They felt that being part of the group was empowering and reduced the sense of stigma surrounding bipolar disorder. Facilitators’ accepting attitude towards them and the humour within the groups was important for “normalising” the illness, and participants reported feeling less isolated and lonely through meeting others “in the same boat”. A few participants compared themselves to others within the group to assess how well they were coping, and felt that it was important to have some people of a similar age in a group together. A few also reported that attending the first group meeting felt intimidating as it was the first time they had met others with bipolar disorder.

#### Impact of BEP-Cymru

Although some participants reported that they did not benefit from the groups because they had not attended many sessions, some participants appreciated the course despite already knowing much of the information presented because the course confirmed their previous knowledge and gave them an opportunity to meet and share experiences with others. A number of participants reported making new friends on the course, no longer felt alone with having the disorder, accepted their diagnosis and their responsibility to self-manage their condition, and understood themselves more. Some participants gained insights into the illness, recognised their personal triggers, and felt more confident and stable as a result of the course. Others reported becoming less resistant to taking medication and felt better able to explain their condition.

### Strengths and limitations

The rich qualitative data from this study has provided insights into how patients experienced a new, group-based psychoeducation programme in Wales. The findings relate to the feasibility, acceptability and impact of the programme from participants’ perspectives and present their recommendations for improvement. Male and female participants from South and North Wales were interviewed to explore perceptions of groups in different locations and with different group facilitators, and those who only attended a small number of sessions were interviewed which enabled exploration of their reasons for attrition. When analysing the data we noted that themes did not cluster according to the number of sessions participants attended nor appeared to affect their self-reported satisfaction (or dissatisfaction) across the range of outcomes. Hence, there was variation in the views and experiences of those who had attended the same number of sessions; i.e., low users and high users felt that the intervention was worthwhile and reported that they missed sessions for logistical reasons or because of their mood at the time, and one low and one high user gave similar criticisms of the facilitators’ delivery style. However, the small sample within this study may limit the theoretic generalizability of findings. A larger sample may have revealed differences in responses by gender, type of illness and differing lengths of diagnosis which have not been salient and therefore possible for interpretation within this sample. Furthermore, a larger and more diverse sample may have provided more insights into the feasibility and acceptability of the intervention for those from different backgrounds with a view to improving equal access to services. There was a particularly high proportion of participants of Caucasian ethnicity (98 %) and the majority were currently in employment (60 %) [17], which is unrepresentative of recruiting participants from NHS settings and throughout the UK. Certain barriers to accessing the group sessions were noted in this group, however, such as the time of day and location of the meetings.

The flexible and responsive interviewing style enabled participants to discuss aspects of their experiences which were relevant to them, and minimised the risk of biasing participants’ responses. This study may have benefitted from having a portion of the data double-coded for reliability, and also from interviewing those who never attended a session. Furthermore, additional follow-up interviews at one year may have provided insight into how participants’ perceptions of bipolar disorder change over time and the longer-term impact of the intervention.

### Clinical implications

Group psychoeducation sessions may be better received in a community-based venue as opposed to a hospital or university venue. Hospitals had distressing connotations for participants, which they felt to be inappropriate settings for the course.

We found that participants’ high or low mood sometimes affected their engagement with BEP-Cymru and Beating Bipolar, so that they may have found it hard to concentrate on the materials or lacked sufficient energy or motivation to engage. Some participants experienced depression during BEP-Cymru, which may not have been a result of the course itself (although confronting their illness may have been a contributing factor), but highlights the importance of support from mental health professionals when undertaking psychoeducation for bipolar disorder and the life chart exercise in particular.

As recommended by many BEP-Cymru participants, it would be helpful for some group psychoeducation sessions to be delivered specifically for partners, relatives and carers to enable them to better understand bipolar disorder and offer support. Furthermore, it may be helpful for medically trained facilitators to recognise the value of an experiential focus from participants’ perspectives, in addition to a clinical focus, and sessions may benefit from less didactic teaching and more discussions.

As participants of group psychoeducation facilitated by mental health professionals, patients may become more knowledgeable about medication for bipolar disorder and the treatment options available to them. Consequently, patients may become less resistant and more confident to experiment with medication or commit to take their medication regularly as prescribed. Psychoeducation may also increase patients’ acceptance of bipolar disorder, reduce perceived social stigma, and enable them to feel more compassionately towards themselves and reinterpret aspects of their life and illness.

Some people with bipolar disorder internalise prejudicial beliefs and emotions to the effect that stigma becomes a barrier to their effective treatment [18]. As with previous findings [9], the present research found that stigma was a pervasive theme amongst patients undertaking psychoeducation, as many had concerns about meeting with others who had bipolar disorder, labelling bipolar disorder as a mental illness rather than a condition or being misunderstood by families or colleagues. Those involved in the care of people with bipolar disorder may consider the finding that some patients may be reluctant to perceive themselves as having an illness, and therefore sensitively refer to bipolar disorder as a “condition”.

## Conclusions

This is the first in-depth qualitative study of patients’ perspectives and experiences of a UK-based group psychoeducation programme for people with bipolar disorder. This research demonstrates that group psychoeducation may impact on participants’ perceived social support, knowledge and acceptance of bipolar disorder, personal insights, attitude towards medication and access to services.

Participants pointed out the importance of informal elements of the group sessions, reporting that that they benefited from learning from others, and consequently felt less lonely and isolated. Meeting other people with bipolar disorder normalised their view of themselves, provided a feeling of solidarity and was de-stigmatising. They appreciated sensitive, flexible and authentic facilitators who were keen to hear their points of view, rather than talking from PowerPoint slides. Therefore, the study provides us with an important complimentary view that psychoeducation should not operate within a too rigid medical framework where participants are being patronized and talked “at” rather than being talked “with”. Psychoeducation group facilitators should have the skills to build group rapport through a group atmosphere where participants may feel at ease, be able to get to know each other, trust the group, share their experiences and have a relatively relaxed and enjoyable time doing so.

The key recommendations presented for improving the content and delivery of group psychoeducation for bipolar disorder may enhance engagement and widen access to such programmes. Future research into psychoeducation for bipolar disorder may explore how to target and engage people of diverse ethnic backgrounds, men and those in lower socioeconomic groups who are less likely to access healthcare services.

## Abbreviations

BEP-Cymru: Bipolar Education Programme Cymru
MDF: Manic Depression Fellowship
